# Photocatalytic Activity and Filtration Performance of Hybrid TiO_2_-Cellulose Acetate Nanofibers for Air Filter Applications

**DOI:** 10.3390/polym13081331

**Published:** 2021-04-19

**Authors:** Miyeon Kwon, Juhea Kim, Juran Kim

**Affiliations:** 1 Human Convergence R&D Group, Korea Institute of Industrial Technology, Ansan 15588, Korea; mykwon@kitech.re.kr (M.K.); juheakim@kitech.re.kr (J.K.); 2 Advanced Textile R&D Group, Korea Institute of Industrial Technology, Ansan 15588, Korea

**Keywords:** filtration efficiency, photocatalyst, titanium dioxide, emulsion electrospinning, cellulose acetate nanofibers

## Abstract

A facile method to prepare hybrid cellulose acetate nanofibers containing TiO_2_ (TiO_2_-CA nanofibers) by emulsion electrospinning technique was developed for the denitrification and filtration of particulate matters (PMs). This work found that hybrid TiO_2_-CA nanofibers mainly contain the anatase form of TiO_2_, contributing to the photodecomposition of NO gas under UV irradiation. The TiO_2_-CA nanofibers also showed an excellent filtration efficiency of 99.5% for PM_0.5_ and a photocatalytic efficiency of 78.6% for NO removal. Furthermore, the results implied that the morphology of the TiO_2_-CA nanofibers, such as micro-wrinkles and protrusions, increased the surface hydrophobicity up to 140°, with the increased addition of TiO_2_ nanoparticles. The proposed TiO_2_-CA nanofibers, as a result, would be promising materials for highly efficient and sustainable air filters for industrial and home appliance systems.

## 1. Introduction

Air pollution is a serious environmental issue, which is continuously burdening our daily lives. In general, the most concerning air pollutants are sulfur dioxide (SO_2_), nitrogen oxides (NO and NO_2_), volatile organic compounds (VOCs), particulate matters (PMs), carbon monoxide (CO), carbon dioxide (CO_2_), ozone, chlorofluorocarbons, and trace heavy metals [1]. Among air pollutants, PMs are comprised of a complex mixture of sulfate, nitrates, ammonia, sodium chloride, carbon black, mineral dusts, and water. Depending on their size, PMs can be classified as PM_0.5_, PM_2.5_, and PM_10_, which denote particle sizes below 0.5, 2.5, and 10 μm, respectively. These PMs are detrimental to human health, since they can penetrate human bronchi and lungs to cause chronic pulmonary disease and lung cancer [2]. In 2013, the World Health Organization also reported that air pollutants such as PMs are carcinogens to humans and can even induce death [3].

Electrospun nanofibers that remove air pollutants such as PMs and VOCs have been of great interest due to their highly specific surface area, interconnected nanoscale pore structures, nanosized fiber diameters, and porous structure, as well as their versatility for incorporating chemical modifications for air filter applications [4]. Particularly, in many applications, such as biomedical or cosmetic products and air filters [5,6], cellulose nanofibers have received increasing attention due to the advantages of large specific surface area, versatile chemical modifications, and good mechanical properties [7].

For a few years, their photocatalytic activities have been well studied for removing various pollutants, such as PMs, SO_2_, NO, NO_2_, and various VOCs, efficiently in air environments [8]. Particularly, photocatalytic degradation using titanium dioxide (TiO_2_) has been one of the most generally studied methods, because it powerfully converts rich solar energy into active chemical energy that can decompose harmful pollutants in the air [9]. TiO_2_ excites electrons under UV illumination from the valence band to the conduction band and leaves holes in the valence band. At that moment, the electrons change oxygen molecules to superoxide anions and the holes react with water molecules in the air to produce hydroxyl radicals. These two species, superoxide anions and hydroxyl radicals, are very reactive and capable of decomposing air pollutants such as PMs, SO_2_, NO, NO_2_, and VOCs [10].

Several TiO_2_ incorporated nanofibers have been of great interest for the efficient removal of air pollutants, because of their well-defined dimensions, high specific surface areas, and greater photocatalytic activities [11,12,13]. For example, polyacrylonitrile (PAN) nanofibers with embedded commercial photocatalysts, P25 and TiO_2_ particles, showed an excellent filtration efficiency of 96.75% for PM_2.5_ [14]. Dong et al. studied effective strategies for in situ growth of high adhesion TiO_2_ to polyvinylidene fluoride (PVDF) nanofibers via electrospinning, coupled with cold plasma pretreatment and hydrothermal processing [15]. On the other hand, bamboo cellulose acetate fibers grafted with TiO_2_ have presented the photocatalytic ability to decompose phenol under UV illumination [16]. Lastly, electrospun TiO_2_ entrapped-chitosan hybrid nanofibers were developed for the removal of heavy metal ions [17].

However, there are some major problems that restrict the application of photocatalytic fibers using TiO_2_. First is the brittleness of TiO_2_ incorporated nanofibers. Li et al. have reported brittle polycrystalline TiO_2_-incorporated nanofibers produced from a precursor solution, such as titanium alkoxide (Ti(OR)_4_) with poly(vinyl pyrrolidone), leading to reduced photocatalytic activities until after calcination [18]. Another problem is that the number studies on the application of TiO_2_-incorporated nanofibers and their photocatalytic activities for simultaneous denitrification has not been sufficient thus far. The weak adhesion between TiO_2_ and the polymeric nanofibers is also a key obstacle that inhibits their practicability. Moreover, the high photocatalytic activity of TiO_2_-incorporated nanofibers, without the degradation of polymeric substrates, remains a critical challenge.

In order to overcome these drawbacks of TiO_2_-incorporated cellulose nanofiber applications, we designed a fast and facile fabrication method for hybrid cellulose acetate nanofibers containing TiO_2_ (TiO_2_-CA nanofibers), by using emulsion electrospinning to reduce weak adhesion and brittleness between TiO_2_ and the polymeric nanofibers. There is the need for a detailed investigation of the photocatalytic effects of TiO_2_ incorporated into nanofibers for the removal of NO gas. In this study, we present a fast and facile fabrication method for hybrid cellulose acetate nanofibers containing TiO_2_ (TiO_2_-CA nanofibers), by using emulsion electrospinning, and evaluate the photodecomposition of nitrogen oxides under UV illumination, as well as the filtration efficiency.

## 2. Materials and Methods

### 2.1. Materials

Titanium isopropoxide (TTIP), cellulose acetate anhydrous sodium sulfate (CA, Mn = 30,000 Da, the degree of acetylation: 39.3–40.3 wt%), isopropyl alcohol (>99.7% grade), acetone, *N*,*N*-dimethylacetamide, NaOH (0.1 M in water), sodium chloride and acetone were purchased from Sigma Aldrich Co., LLC, Korea (Seoul, Korea).

### 2.2. Synthesis of TiO_2_ Nanoparticles

10 mL of the titanium isopropoxide (TTIP) as a precursor was mixed with 40 mL isopropyl alcohol and stirred for 30 min. Then 10 mL of a mixture (1:1) of deionized water and isopropyl alcohol was added gradually in drops into the TTIP mixture to form a colloidal solution under vigorous stirring. The pH of the obtained colloidal solution was adjusted using NaOH solution and irradiated by sonication (Power sonic 510, Seoul, Korea) at 40 kHz for 30 min. The colloidal solution was dried in an oven at 110 °C for 3 h and TiO_2_ nanoparticles were calcinated at 400 °C for 1 h.

### 2.3. Fabrication of TiO_2_-CA Nanofiber Webs

TiO_2_-CA nanofibers were fabricated using emulsion electrospinning. First, 10 wt% CA pellets were dissolved in a mixture of *N*,*N*-dimethylacetamide and acetone at the ratio of 1:2 (*v*/*v*). The solution was then stirred at 500 rpm and room temperature for 5 h. Afterwards, 5 or 10 wt% of synthesized TiO_2_ nanoparticles of CA pellets were put into the CA solution. To evenly disperse TiO_2_ nanoparticles in CA solutions, sonication (Power sonic 510, Seoul, Korea) was employed at 40 kHz for 30 min. The solution was put into a 15 mL syringe and electrospun at a feeding rate of 1 mL h^−1^ on the roller collector, covered with a melt-blown polyester nonwoven supporter. The distance between the Taylor cone and the collector was 15 cm, and 18 kV was applied for the fabrication of TiO_2_-CA nanofiber webs.

### 2.4. Characteristics of Synthesized TiO_2_ Nanoparticles and TiO_2_-CA Nanofibers

An X-ray diffractometer (XRD, D8 Advance, Bruker, Billerica, MA, USA) was used to analyze crystalline phases of the synthesized TiO_2_ nanoparticles and TiO_2_-CA nanofibers. The XRD was operated in reflection mode with Cu-K radiation (35 kV, 30 mA) and diffracted beam monochromator, using a step scan mode with a step of 0.075° and 4 s per step. Diffraction patterns of both anatase and rutile TiO_2_ powders were compared with references in the Joint Committee on Powder Diffraction Standards (JCPDS) database.

### 2.5. Contact Angle Measurement and Morphology of TiO_2_-CA Nanofiber Webs

The contact angle of TiO_2_-CA nanofiber webs was measured using a contact angle goniometer (DSA 25, Kruss, Matthews, NC, USA) and the sessile drop technique at room temperature. Then, 10 μL of deionized water (γ_LV_ = 72.8 mN/m) droplet was deposited on CA and TiO_2_-CA nanofiber webs using a syringe, and the measurement was repeated at least five times in order to analyze the hydrophobicity of the TiO_2_-CA nanofiber webs. Additionally, the morphology and average diameter of TiO_2_ nanoparticles, untreated CA nanofibers, and TiO_2_-CA nanofibers were analyzed by scanning electron microscopy (SEM, Hitachi, Tokyo, Japan), with an energy dispersive X-ray analyzer (EDX) to evaluate the atom weight percentage on the surface of the TiO_2_-CA nanofibers.

### 2.6. Photocatalytic Activity of TiO_2_-CA Nanofiber Webs for NO Removal

The photocatalytic activities of TiO_2_-CA nanofiber webs were evaluated based on ISO 22197-1:2016 for the removal of NO gas. TiO_2_-CA nanofiber or untreated CA nanofiber (5 cm × 10 cm) samples were placed in the middle of two plain glasses (5 cm × 10 cm) of non-photocatalytic blank samples in the photoreactor. A UV lamp system was placed over the photoreactor, and delivered a UVA irradiance (10 W m^−2^) from 2 × 6 W BLB lamps with a 365 nm emission peak. All three samples were illuminated with UV light. NO_x_ analyzer (T-API, T200, San Diego, CA, USA) was used to measure nitrate concentrations every 1 min. NO gas flowed at a rate of 3 L min^−1^ containing 1 ppm_v_ of NO in air with 50% of relative humidity at 25 °C under UV light. The concentration of NO in the outlet stream was monitored for 20 min before the light was switched on, and then during the 1 h UV irradiation.

### 2.7. Filtration Efficiency of TiO_2_-CA Nanofiber Webs

For filtration efficiency and penetration (%), TiO_2_-CA nanofiber webs were measured using a TSI-3160 filter tester (TSI Inc., Shoreview, MN, USA). In this system, sodium chloride particles were used as representative PMs. This tester was able to generate sodium chloride nanoparticles with sizes ranging between 100 and 600 nm in the flowing air, and measuring particle penetration versus particle size at 32.28 L min^−1^ aerosol flow rate and 5.38 cm s^−1^ face velocity. TiO_2_-CA nanofiber webs were placed at the bottom of a sample holder on a wide-mesh metal net to support the specimen. The percentage penetration of sodium chloride particles passing through the TiO_2_-CA nanofiber webs was determined by the relative particle concentration upstream and downstream of the sample [19].

## 3. Results and Discussion

### 3.1. The Morphology of TiO_2_ and TiO_2_-CA Nanofibers

**Figure 1:**
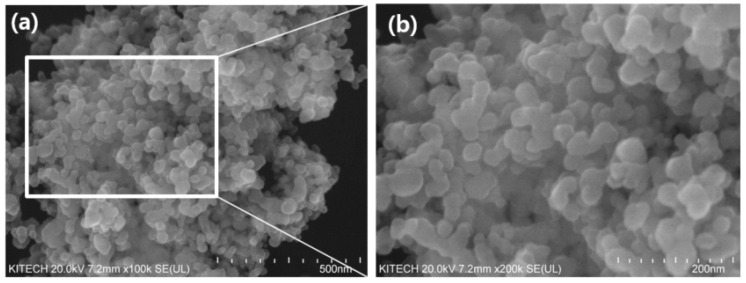
The morphology of (**a**) the synthesized TiO_2_ nanoparticles (**b**) the enlarged image of the square on the left.

Figure 1 shows the morphology of the synthesized TiO_2_ nanoparticles, which displayed an irregular shape and rough surface without pores. The diameters of the TiO_2_ nanoparticles were measured from randomly selected areas of SEM images using Nahwoo imaging software (N ≥ 100) (Iworks 2.0, Suwon, Korea). The average diameter of the TiO_2_ nanoparticles was 54 nm (±5.6).

**Figure 2:**
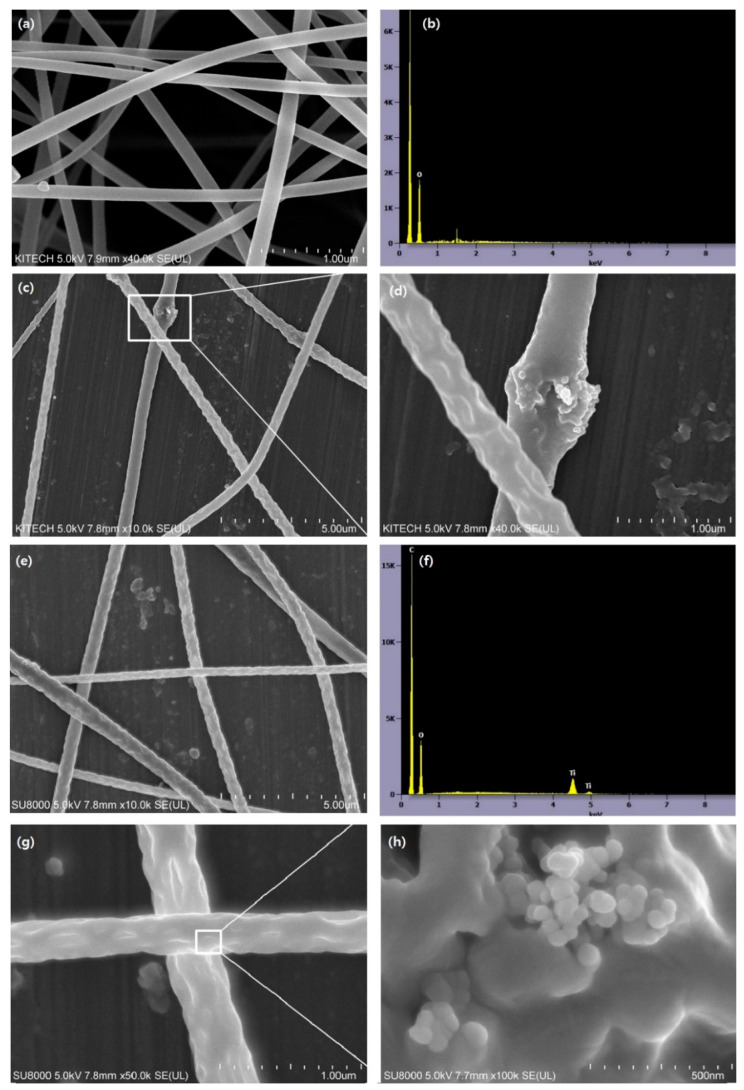
SEM images of (**a**) untreated CA nanofibers, (**b**) EDX analysis of untreated CA nanofibers, (**c**) 5 wt% TiO_2_-CA nanofibers, (**d**) the enlarged image of the square on the left, (**e**) 10 wt% TiO_2_-CA nanofibers, (**f**) EDX analysis of 10 wt% TiO_2_-CA, (**g**) the surface morphology of 10 wt% TiO_2_-CA nanofibers, and (**h**) the enlarged image of the square on the left.

As shown in Figure 2, the untreated CA nanofibers had smooth surfaces and no defects. The average diameter size of the CA nanofibers was 278 nm, with a standard deviation of 78 nm; however, the TiO_2_-CA nanofibers displayed increasing roughness and average diameter size with the addition of TiO_2_ loadings. CA nanofibers containing 5 or 10 wt% TiO_2_ induced the formation of abnormal beads on the surface that resulted in an uneven morphology and micro-wrinkles [20]. During electrospinning, a highly volatile solvent can solidify immediately on the surface of the nanofibers while the fluid jet is flying to the collector, whereby it becomes hard for the nanoparticles to be transferred from the core to the shell of the nanofibers [21]. Compared to the diameter range of the 5 wt% TiO_2_-CA nanofibers, from 200 to 1050 nm, the average diameter of the TiO_2_-CA nanofibers was 378 nm (σ = 74), as shown in Figure 2c,d. Figure 2e highlights the 454 nm (σ = 126) average diameter of 10 wt% TiO_2_-CA nanofibers, lying within the 150 to 1300 nm diameter range of 10 wt% TiO_2_-CA nanofibers. The rough surface and grafted TiO2 nanoparticles are shown in Figure 2g,h. In Figure 2b,f quantitative analyses of each untreated CA nanofiber element and 5 and 10 wt% TiO_2_-CA nanofibers are compared through an energy dispersive X-ray analyzer (EDX, attached to the SEM). The data show spectra with peaks corresponding to all the different elements. In Table 1, the EDX data show each element concentration percentage for the different samples. For 5 wt% TiO_2_-CA nanofibers, carbon and oxygen atoms mainly occupied 47.1 wt% and 46.7 wt% of the sample, respectively. Untreated CA nanofibers exhibited no Ti content on the surface. In contrast, 5 and 10 wt% TiO_2_-CA nanofibers comprised 6.3% and 11.6 wt% Ti on the surface, respectively. From the data, we confirmed that the final TiO_2_-CA nanofibers maintained a similar weight to the initial addition of TiO_2_.

### 3.2. XRD Analysis of TiO_2_ Nanoparticles and TiO_2_-CA Nanofibers

**Figure 3:**
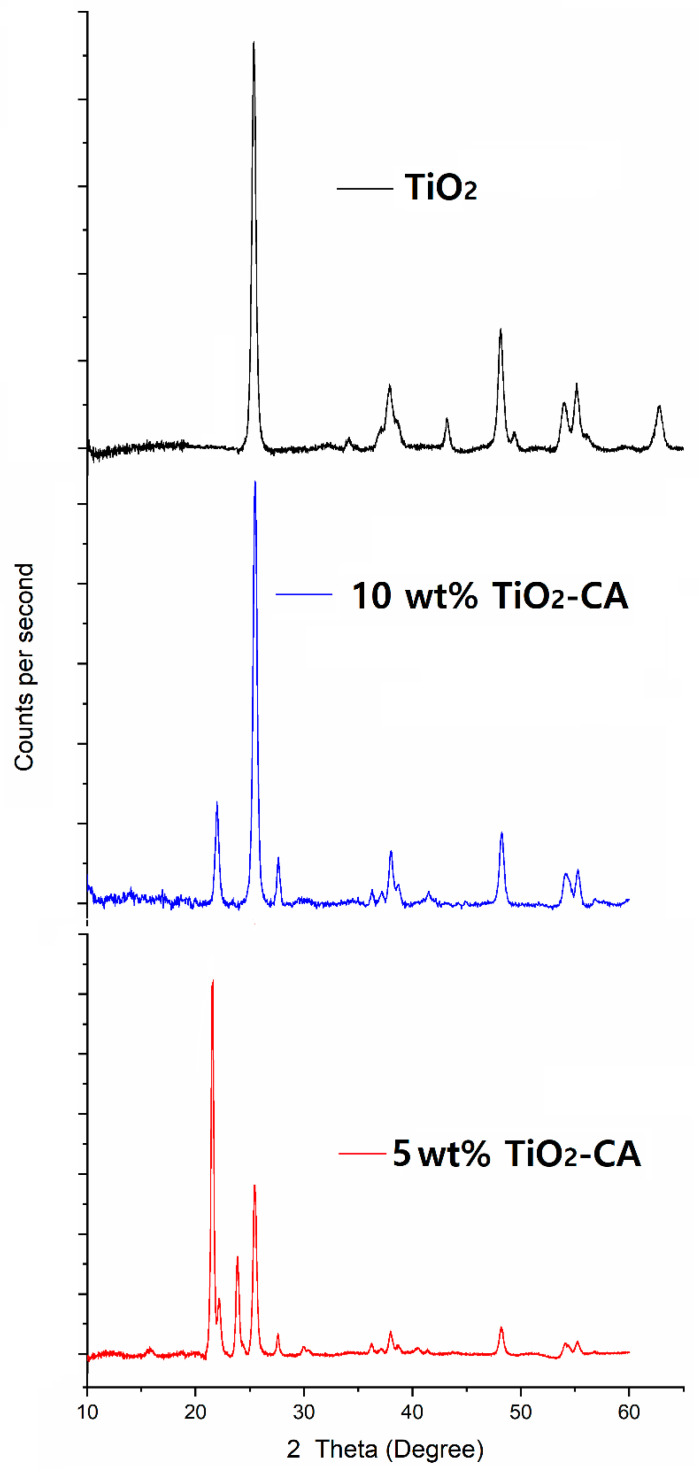
XRD patterns of synthesized TiO_2_ nanoparticles, and 5 and 10 wt% TiO_2_-CA nanofibers.

Both anatase and rutile are well known as stable phases of TiO_2_ nanoparticles [12]. TiO_2_ nanoparticles are most likely to be a mixture of those phases, rather than pure anatase or rutile; therefore, quantitative analysis is highly important. Figure 3 and Table 2 show the XRD patterns of the synthesized TiO_2_ nanoparticles and TiO_2_-CA nanofibers in the 2θ range of 10–70°, according to standard JCPDS card No. 21-1272. The anatase reflections dominated the reflection patterns, while rutile was present as well. All diffraction peaks at 25.25°, 37.80°, 38.50°, 48.05°, 53.9°, 55.05°, 62.65°, 68.85°, 70.30°, 75.05°, and 76.10° were well indexed as pure anatase phases. Rutile phases showed diffraction peaks at 27°, 36°, and 55°, the crystalline region of TiO_2_ [22]. For 5 or 10 wt% TiO_2_-CA nanofibers, the new diffraction peaks at 22.6° mainly represented the crystalline region of the cellulose [16,23]. From the XRD analysis, the results show that crystalline TiO_2_ nanoparticles consisted of 87.8% anatase and 12.7% rutile forms, and TiO_2_-CA nanofibers contained both anatase and rutile forms of TiO_2_ nanoparticles after fabrication.

### 3.3. The Photocatalytic Effect of NO Removal

**Figure 4:**
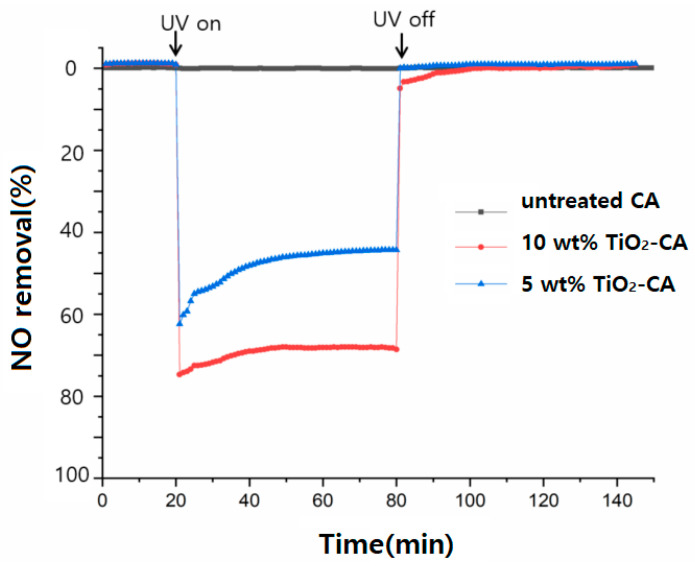
The photocatalytic effect of TiO_2_-CA nanofibers for NO gas removal.

In order to confirm the inevitability of UV irradiation for photocatalytic reaction, tests of UV irradiation (turning on UV lamp) and darkness (turning off UV lamp) for denitrification were carried out, as exhibited in Figure 4.

The results show that the NO removal efficiency changed greatly with or without UV irradiation. Specifically, the denitrification efficiency in the dark was less than 0.1%, mainly due to the physical adsorption of NO over TiO_2_-CA nanofibers. After turning on the UV light for 60 min, the NO removal efficiency of 5 or 10 wt% TiO_2_-CA nanofibers showed a rapid upward trend and increased to 64.5% and 78.6%, respectively. Untreated CA nanofibers, however, showed no effect with or without UV light.

When the UV light was turned off, the removal efficiency of NO decreased rapidly to the initial adsorption equilibrium level in the dark. This implies that there is a significant effect of UV irradiation on NO removal, meaning that UV light is a vital factor in the photocatalytic reaction of NO gas removal.

### 3.4. The Filtration Efficiency of TiO_2_-CA Nanofiber Webs

**Figure 5:**
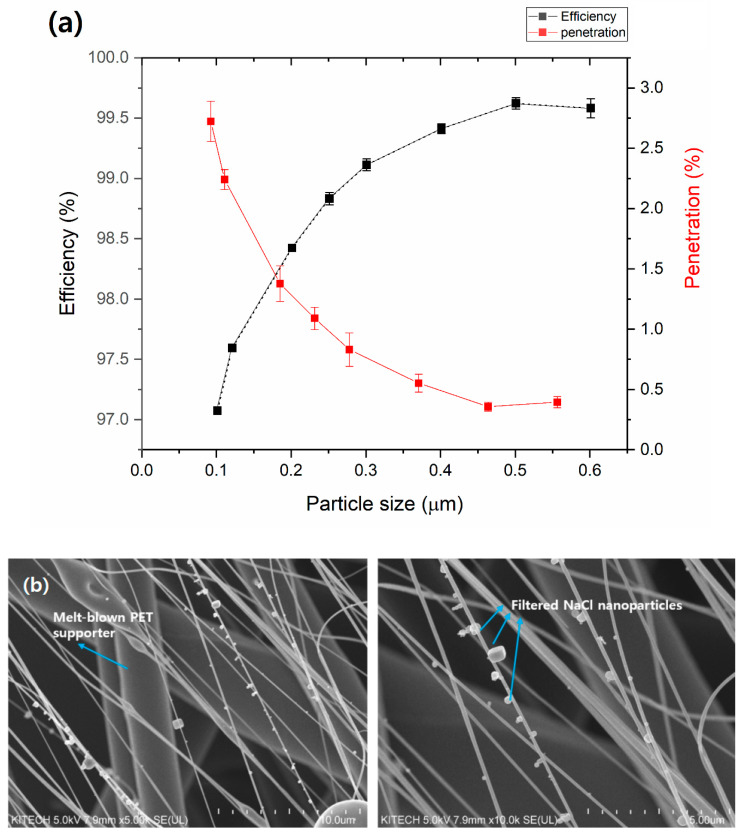
(**a**) Diagrams of the filtration efficiency (%) and air penetration (%) of 10 wt% TiO_2_-CA nanofiber webs, and (**b**) captured NaCl particles (represented as PMs) on TiO_2_-CA nanofibers.

Various sizes of PMs were tested for filtration efficiencies of 10 wt% TiO_2_-CA nanofiber webs. Figure 5a shows the filtration efficiency (%) and penetration (%) of 10 wt% TiO_2_-CA nanofibers, depending on NaCl particle size. Clearly, the filtration efficiencies against all sizes of PM were higher than 97%. TiO_2_-CA nanofiber webs showed increased efficiencies from 97.1 to 99.6% in particle sizes 0.1 (PM_0.1_) to 0.6 µm (PM_0.6_). However, penetration decreased from 2.93 to 0.42%. The pressure drop was consistently near 3.13 mm H_2_O for most particle sizes.

SEM images of 10 wt% TiO_2_-CA nanofibers after capturing NaCl nanoparticles (represented as PMs) are shown in Figure 5b. Some 0.1–0.6 µm sized PMs were captured at the surface and around nanofibers, revealing a strong electrostatic force was present at the surface of 10 wt% TiO_2_-CA nanofibers, which attracted PMs that are much smaller than the thru-holes because of the electrostatic force created by air flow friction. A study on the electrosurface properties and interaction of cellulose nanofibers and TiO_2_ nanoparticles stated that cellulose nanofibers and titanium dioxide nanoparticles were attracted to one another due to electrostatic forces on the surface [24].

### 3.5. Contact Angle Analysis

**Figure 6:**
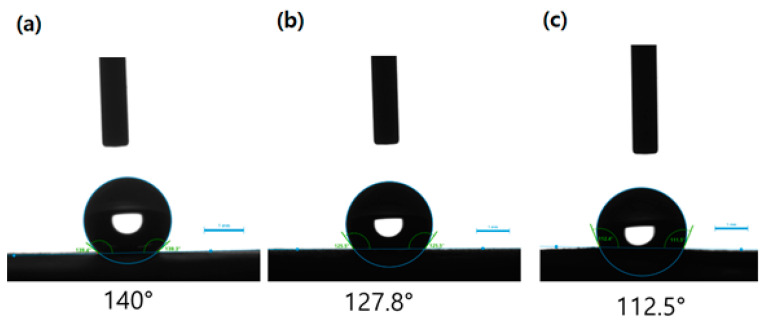
Contact angles of (**a**) 10 wt% TiO_2_-CA nanofibers, (**b**) 5 wt% TiO_2_-CA nanofibers, and (**c**) untreated CA nanofibers.

The hydrophobicity of untreated CA or TiO_2_-CA nanofiber webs was subsequently measured, with the results of water contact angle measurements from different samples illustrated in Figure 6. In all membranes incorporated with TiO_2_ nanoparticles, the value of the contact angles increased with the addition of TiO_2_ loadings. To be specific, the contact angle of untreated CA nanofibers was 112.5°, while that of TiO_2_-CA nanofibers increased from 127.8° to 140° with the addition of TiO_2_ nanoparticles. When TiO_2_ nanoparticles were incorporated with CA nanofibers, the hydrophobicity of TiO_2_-CA nanofibers improved due to the surface roughness, as shown in Figure 2g,h. This supports previous research indicating an improved surface hydrophobicity that stems from morphology traits such as micro-wrinkles and protrusions of nanofibers through the mitigation of wetting and decreasing water-membrane contact area [25]. Guan also studied the relationship between hydrophilicity and photocatalytic activity: a surface with more hydrophilicity resulted in less photocatalytic activity [26]. When photocatalysts included a hydrophobic surface with a nano- or microstructure, it enhanced photocatalytic activities [27]. Since the charges on the hydrophobic surface are transferred quickly to radicals by acceptors (oxygen molecules), oxygen molecules can effectively capture electrons and change oxygen molecules to superoxide anions, which can be attributed to photocatalytic activities [28,29]. Due to these fast transitions to radicals on a more hydrophobic surface, 10 wt% TiO_2_-CA nanofibers showed a higher NO removal, up to 78.6%, than 5 wt% TiO_2_-CA nanofibers. A hydrophobic surface also presents a strong electrostatic force by air flow friction that attracts more PMs to the surface. In this work, the greater hydrophobic surface of 10 wt% TiO_2_-CA nanofibers could cause a stronger interaction between filters and PMs than 5 wt% TiO_2_-CA nanofibers, leading to the removal of air pollutants.

## 4. Conclusions

In this study, we have shown a facile and efficient fabrication method to prepare hybrid TiO_2_-CA nanofibers for photocatalytic denitrification activity and the improved filtration of PMs, and evaluated the photodecomposition of nitrogen oxides under UV illumination, as well as the filtration efficiency for the removal of air pollutants.

The photocatalytic effects of TiO_2_-CA nanofiber denitrification decomposed NO gas up to 78.6%. A single-layer TiO_2_-CA nanofiber web effectively filtered and captured PMs (0.1–0.6 µm) up to 99.6%. In addition, further investigations on the possible mechanism between the filter performance and the roughness of the nanofiber filter, as well as the photocatalytic ability to decompose other VOCs, such as toluene, SO_2_, and formaldehydes under ultraviolet irradiation are required. As a result, this investigation demonstrates a novel method of fabricating TiO_2_-CA nanofibers and utilizing their high photocatalytic efficiency for a wide range of potential applications, including protective clothing systems, sensors, industrial and home appliance filtration systems, and much more. TiO_2_-CA nanofibers are a promising natural alternative to replace synthetic polymers in air filter applications.

## Figures and Tables

**Table 1:** Quantitative analysis of untreated CA and TiO_2_-CA nanofibers.

Materials	5 wt% TiO_2_-CA Nanofibers		10 wt% TiO_2_-CA Nanofibers		Untreated CA Nanofibers	
Element	Weight %	Atom %	Weight %	Atom %	Weight %	Atom %
C	47.1	56.2	42.7	53.4	49.5	56.6
O	46.7	41.9	45.7	42.9	50.5	43.4
Ti	6.3	1.9	11.6	3.6	-	-

**Table 2:** XRD analysis of synthesized TiO_2_ nanoparticles and TiO_2_-CA nanofibers.

Sample	Compound	2θ (°)
TiO_2_ Nanoparticles	Anatase	25.25°, 37.80°, 38.50°, 48.05°, 53.9°, 55.05°, 62.65°
	Rutile	27°, 36°, 55°
TiO_2_-CA nanofibers	Cellulose	22.6°
	Anatase	25.25°, 37.80°, 38.50°, 48.05°, 53.9°, 55.05°
	Rutile	27°, 36°, 55°
